# Experimental Verification on Steering Flight of Honeybee by Electrical Stimulation

**DOI:** 10.34133/2022/9895837

**Published:** 2022-07-21

**Authors:** Li Yu, Jieliang Zhao, Zhiyun Ma, Wenzhong Wang, Shaoze Yan, Yue Jin, Yu Fang

**Affiliations:** ^1^ School of Mechanical Engineering, Beijing Institute of Technology, Beijing 100081, China; ^2^ Division of Intelligent and Biomechanical Systems, State Key Laboratory of Tribology, Department of Mechanical Engineering, Tsinghua University, Beijing 100084, China; ^3^ Institute of Apicultural Research, Chinese Academy of Agricultural Science, 100193, China

## Abstract

The artificial locomotion control strategy is the fundamental technique to ensure the accomplishment of the preset assignments for cyborg insects. The existing research has recognized that the electrical stimulation applied to the optic lobes was an appropriate flight control strategy for small insects represented by honeybee. This control technique has been confirmed to be effective for honeybee flight initiation and cessation. However, its regulation effect on steering locomotion has not been fully verified. Here, we investigated the steering control effect of honeybee by applying electrical stimulation signals with different duty cycles and frequencies on the unilateral optic lobes and screened the stimulus parameters with the highest response successful rate. Moreover, we confirmed the effectiveness of steering control by verifying the presence of rotation torque on tethered honeybees and the body orientation change of crawling honeybees. Our study will contribute some reliable parameter references to the motion control of cyborg honeybees.

## 1. Introduction

Miniature air vehicle (MAV) plays an indispensable role in a wide range of military applications such as tracking [1, 2], reconnaissance [3], and relay communication [4, 5]. For decades, MAV has received considerable critical attention and has synchronously developed in two fields, including microaircrafts based on bionic design and cyborg insects whose locomotion are controlled by artificial stimulation modules. Due to inheriting the senor organs and athletic ability of the insect carriers, cyborg insects remedy the technical limitations of bionic aircrafts in terms of deficient sensing equipment [6, 7], poor adaptability in an unstructured environment [8], and difficulty in continuous power supply [9]. Moreover, cyborg insects have incomparable application potential in biodiversity protection.

A high reliable, fast responsive, and minimal destructive flight control strategy is the pivotal technology to ensure the cyborg insect to complete the preset assignments. According to the sites for stimulation, the flight control strategies for insects are classified as flight muscle stimulation [10–12], sensory organ stimulation [13–15], and neuron stimulation [16–19]. Among them, the control strategy based on flight muscle electrical stimulation has a widespread application on large insects represented by beetles and moths [10, 20–23]. The artificial control of flight initiation and prohibition has been achieved by the pulse electrical stimulation on dorsal longitudinal muscle of the beetles [24]. Moreover, their flapping elevation angle, flapping amplitude, and flight speed can be modulated by the stimulation on basalar muscle [11, 21], 3Ax muscle [20, 25], and subular muscle [11]. The existing research has demonstrated that the control of multiple flight parameters for insects requires simultaneous electrode implantation and electrical stimulation on more than one flight muscles. However, the strategy of multichannel muscular electrical stimulation is undoubtedly more challenging for small invertebrates like honeybee and *Drosophila*, because of their more exquisite and sophisticated flight muscle composition. From the perspective of the impact on the biological tissues, the stimulation applied to the muscles will inevitably lead to permanent tissue damage. However, the damage on biological tissue will be significantly reduced by employing neurotransmitter to instead electrical stimulation. Inhibitory neurotransmitter *γ*-aminobutyric acid, for instance, leads to a temporal relaxation of beetle leg muscles [26, 27]. Nevertheless, the neurotransmitter frequently induces a torpid locomotion response. Meanwhile, the specific locomotion parameters cannot be modulated by neurotransmitter injection as well.

Indistinctive tissue destruction is the significant advantage of locomotion control strategy based on sensory organ stimulation, including antenna/cerci electrical stimulation and compound eye optical stimulation. Among them, antenna and cerci with numerous receptors are widely harnessed as electrical stimulation sites for behavior control such as steering [13, 28–31], accelerating crawling [32], and jumping [33]. Despite favorable control effects and successful rate in the above behavioral control, antenna and cerci stimulation have not yet achieved success in flight regulation on insects. Nevertheless, compound eye optical stimulation has proven to be an effective means of flight control for locusts. Mann et al. have assembled a wearable wireless backpack containing LED lights for optical stimulation and accomplished a flight initiation and ceasing on locust [14]. However, the existing wireless devices designed for optical stimulation usually have large volume and weight, which are not able to satisfy the load capacity requirements of small insects.

Inflicting electrical signal on the neuronal tissue will induce the insects to change their body postures and locomotion states. Taking the conversion process of the insect visual signals as an example, the ambient optical flows received by compound eyes and ocellus are separated and then transmitted into neuroelectric signals through lamina, medulla, and lobula in the optic lobes [34, 35]. The lobula plate tangential cells (LPTCs) presenting in lobula plate have a pivotal role in visual information processing [36, 37]. In Drosophila, for example, the LPTCs overlap the neurites of three descending neurons that receive visual input from different LPTCs. The descending neurons terminate in various thoracic motor neuropils, responding strongly to roll and yaw motions [38]. Subsequently, the motor neurons which overlap in the thoracic neuropils exhibit action potential changes and release neurotransmitters at the terminal synapses to induce the muscles to generate contractile behavioral responses [39, 40]. For insects with asynchronous flight muscles, their flight muscles begin to contract at a much higher frequency than that of the motor neuron action potential, once they were activated [41]. Therefore, electrical stimulation on nerve tissues is a mainstream locomotion control strategy with low power consumption for asynchronous flight muscle insects represented by honeybees. To date, optic lobes have become the one of the main locations for sensory nerve center stimulation [42–45]. Moreover, the large volume of the optic lobes is conducive to reduce the difficulty of experimental operation and maintain the functional integrity. Presently, Zhao et al. have verified the effectiveness of flight initiation of honeybee when the bilateral optic lobes received a pulse electrical signal and furtherly optimized the initiation electrical parameters to acquire a better successful rate [46, 47]. Moreover, the current scheme for steering flight control of honeybee is to apply the optimal electrical parameters adopted by flight initiation to the unilateral optic lobe. However, it has not been confirmed that whether the optimal signals for initiation control can induce an effective turning behavior of honeybee in different locomotion states and obtain an ideal successful rate.

In the paper, we aim at optimizing the pulse electrical stimulation parameters for steering flight control of honeybees based on unilateral optic lobe electrical stimulation. To verify the effectiveness of the induced steering action, we designed a magnetic levitation experimental system to reflect the rotational torque necessary for honeybee to generate a steering flight. Furthermore, the electrical stimulation on the unilateral optic lobe was also confirmed to be effective for steering initiation of crawling honeybees. Our experimental results offer some reliable control parameters for an artificial flight regulation of honeybee and further promote the research of cyborg insect control strategy.

## 2. Materials and Methods

### 2.1. Study Insect

Specimens of *Apis mellifera L*. were employed for experimental operations. The honeybees were bought from Fragrant Hills Park in Beijing, China, and were raised in an indoor glass feeding box. The feeding devices were kept in a room temperature and a humidity of 50%. Only forgers were selected for experiments.

### 2.2. Electrode Implantation

**Figure 1:**
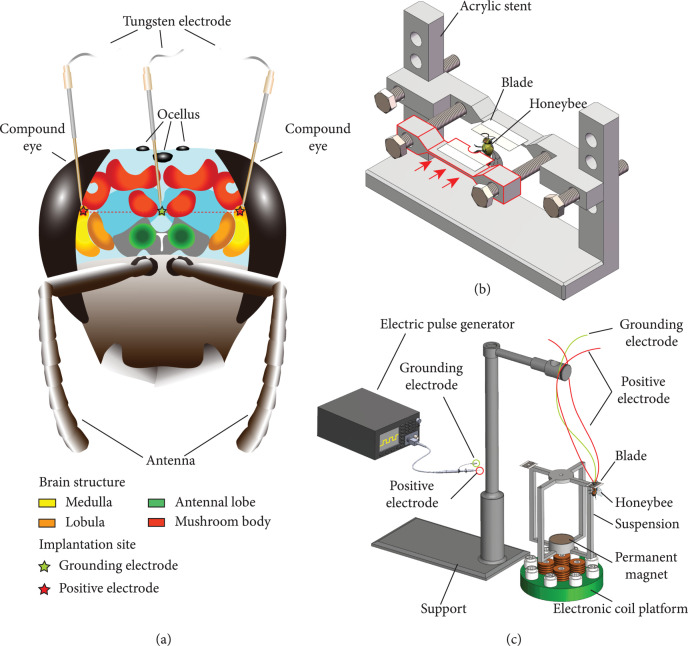
Experimental system for steering flight control and swerving torque verification of honeybees. (a) Implantation sites for stimulating electrodes. (b) Fixation method of the head capsule before electrodes implantation. (c) Experimental system for the verification of the steering torque under stimulation.

The stimulation experiments were performed with tungsten wires (Kedou Brain-Computer Technology Co., Suzhou, China, polyimide-coated, 50 *μ*m diameter). Both ends of the electrodes were burned to remove the insulating coats before being inserted. Prior to the implantation, the honeybees were placed into refrigerator for cryoanesthesia and fixed on the acrylic stent soon afterwards. The neck of the honeybee was immobilized by the two semicircular holes on the distal part of the blades (Figure 1(b)). Subsequently, the head was further immobilized by the beeswax. To facilitate electrode implantation, three holes were firstly poked on the head capsule by the insect needle. The locations of the holes corresponded to the optic lobes on double sides and the midpoint of the central line of the brain (Figure 1(a)). The central line of the brain was conveniently determined by connecting the ocellus and the midpoint of the two antennae. The distance between the holes above the optic lobes and the hole in the center was 906 *μ*m [48]. After the capsule was punched, two tungsten wires working as stimulating electrodes were implanted into the holes on left and right sides, and one tungsten wire serving as grounding electrode was implanted into the middle hole. The implantation depths of stimulating and grounding electrodes were 805 *μ*m and 400 *μ*m from the head capsule, respectively. The localization of the insertion points and the implantation of the electrodes were manipulated using stereotaxic apparatus (SA-151, Yuyan Instruments, China). The electrodes were immobilized by dental glue (zinc polycarboxylate cement, Dental Medicine Material Factory Hospital of Stomatology Wuhan University School) after implantation. The other ends of the wires were connected to the electric pulse generator (33500B, Keysight, China).

### 2.3. Determination of the Optimal Parameters for Steering Flight Stimulation

The honeybees were lifted and suspended to avoid autonomous locomotion after the electrodes were tightly immobilized. We kept the head of the individual vertical and upward, so as to observe the behavioral response under stimulation. The steering flight was recorded by a high-speed camera (Memrecam HX-7s, NAC, Japan) at 3000 frames per second. A pulse electrical signal with 3 V in amplitude, 100 Hz in frequency, 40% in duty cycle, and 1 s in duration was adopted as the benchmark parameter in stimulating the optic lobes. The benchmark parameter was quoted from the results with the highest flight stimulating efficiency by Zhao et al. Here, we mainly study the influence of variation in frequency and duty cycle on the efficiency of steering flight stimulation.

On the one hand, we firstly explored the influence of the duty cycle on the steering response. We kept the voltage, frequency, and stimulation duration identical with those of the benchmark parameter and recorded the locomotion response of each honeybee under the electrical stimulation with duty cycles of 20%, 30%, 40%, 50%, and 60%, respectively. Each electrical signal with different duty cycles was used to initiate a steering flight of one honeybee 20 times, including 10 times on the left optic lobe and 10 times on the right optic lobe. The stimulation sequences on five honeybees were different, so as to reduce the effect of impaired activity of individuals.

On the other hand, we further investigated how the frequency of pulse electrical signal affected the steering response of honeybees. The voltage amplitude and the duration of stimulation parameters we adopted here were consistent with those of the benchmark parameter. However, the duty cycle originated from the value with the highest steering stimulation efficiency acquired from the above experiments. Here, the electrical stimulation signals with 10 Hz, 50 Hz, 100 Hz, 150 Hz, and 200 Hz in frequency were applied to 5 individuals. Moreover, the sample size and the sequence rules for applying each electrical stimulation here were in accordance with those in the previous step.

### 2.4. Verification of the Steering Torque under Stimulation

After obtaining the optimal stimulation parameters for steering initiation of honeybees, we established a magnetic levitation experimental system to verify the swerving torque indispensable for a steering locomotion while changing the body posture, so as to confirm the effectiveness of steering initiation. The experimental system is shown in Figure 1(c), consisting of a platform with four electronic coils, a permanent magnet, blades, an electric pulse generator, a support, and a suspension for immobilizing the magnet and blades. The four electronic coils on the platform were connected in series, so each two coils generated opposite magnetic fields after being energized. The opposite magnetic fields applied thrust and tension to the suspended permanent magnet. The component forces of the tension and the thrust offset each other on the horizontal plane and offset the total gravity of suspension and permanent magnet, so as to maintain a force balance of the suspension and the magnet within 2-3 cm above the electronic coil platform. The resinous suspension was designed into a symmetrical shape and was processed by 3D printing (printed by Future 8200 Resin, density 1.11~1.15 g/cm^3^, WeNext Technology Co., Shenzhen, China) whose weight was evenly distributed on the four rotating arms. The detailed design principle and the composition of the magnetic levitation system, as well as the dimension of the suspension, are shown in Supplementary Material.

Three tungsten wires used for implantation in Section 2.2 were replaced by copper wires (Yuhao Co., Shandong, China, polyimide-coated, 100 *μ*m diameter). Before implantation, the insulating coats on both end of the wires were burned out. The honeybee with immobilized electrodes was kept on the end of the suspension cantilever through blades. The methods for head and neck immobilization were identical with those in Section 2.2. After fixation operations, the ventral side of the honeybee faced the central axis of the suspension. Subsequently, the middle of the copper wires was locked by the support adjacent the electronic coil platform, and the ends of the wires were connected to the electric pulse generator. The lengths of the copper wires reserved between the head capsule and the fixation point on the support were longer than the linear length between the head capsule and the fixation point, so as to avoid the interference of the copper wires to the suspension stability. Notably, the distance between the electric pulse generator and the electronic coil platform must be more that 20 cm, due to the electromagnetic interference.

Before steering initiation, the suspension was manually stabilized to avoid the rotation and keep stationary. It was confirmed that the swerving torque existed if the suspension rotated while the honeybee performed a steering posture under stimulation.

### 2.5. Steering Stimulation to the Crawling Honeybees

For the purpose of verifying the steering control effect of crawling honeybees under electrical stimulation, honeybees with implanted electrodes in the optic lobes were placed in the center of the circular disk marked with orientations. To minimize the interference of the electrodes on the autonomous movement of honeybees, copper wires with a length of more than 30 cm were harnessed for implantation, and the other ends of the wires were connected to the electric pulse generator. The electrical pulse for steering initiation was derived from the optimal parameters determined in Section 2.3. Five honeybees were used for steering regulation, and each individual was initiated 20 times, including 10 times on the left optic lobe and 10 times on the right optic lobe. The behavior response was recorded with a DSLR camera (D850, Nikon, Japan) at a rate of 60 frames per second.

## 3. Results

### 3.1. Optimal Parameters for Honeybee Steering Flight Stimulation

**Figure 2:**
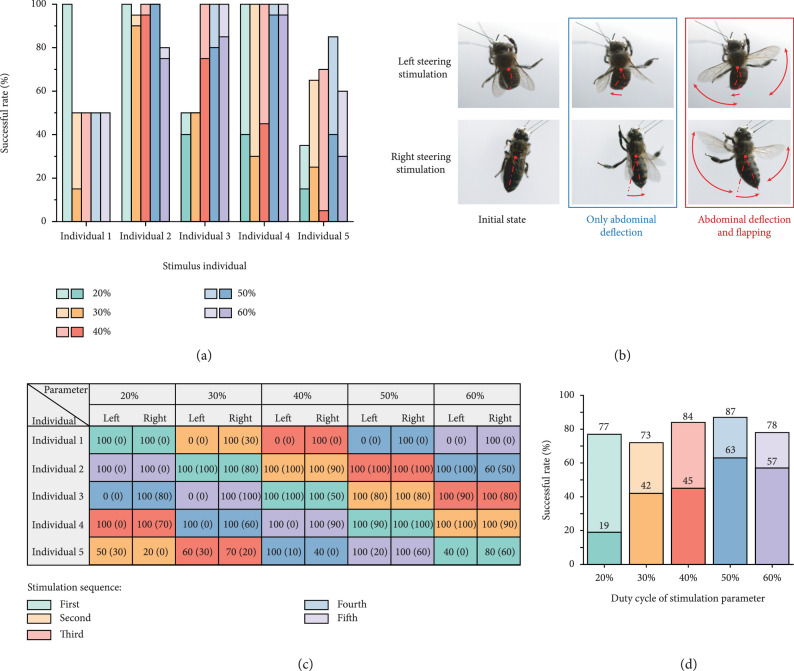
Effective steering responses and successful rates of honeybee under electrical stimulations with different duty cycles. (a) Successful rates of each individual to different electrical stimulus parameters. Among the two representative colors of each duty cycle, the lighter one represents η, and the darker one represents φ. (b) Action feedbacks of honeybees under steering electrical stimulation. (c) The sequence of electrical stimulation received by each individual and the respective successful rate of left and right steering initiation. The ratio outside the brackets is η, and the ratio inside the brackets is φ. (d) The overall successful rates for each stimulus parameter in total five honeybees. In the bar chart of each duty cycle, the upper ratio is η, and the lower ratio is φ.

Honeybees in different locomotion states implement distinct strategies to accomplish a steering behavior. For the free-flying honeybees, the steering posture relies on the combination of abdominal deflection and the change of wing posture [49]. Otherwise, the steering posture of the crawling honeybee mainly depends on the adjustment of the foot locomotion. For the purpose of obtaining the optimal steering control effect, we firstly applied the electrical stimulation with various parameters on the unilateral optic lobe of tethered honeybees. The tethered honeybees produced behavioral responses when received a stimulation on the unilateral optic lobes, including abdominal deflection, wing raise, wing flapping, and wing trembling. Among the behavioral responses, it was found that the abdomen of the honeybee generally deflected to the stimulation side (Figure 2(b)). In addition to abdominal deflection, part of the honeybees raised and flapped their wings asymmetrically. As shown in Figure 2(b), when the left optic lobe received electrical stimulation, the wings on the left side flapped closer to the abdomen, while the wings on the right side flapped closer to the head. Similarly, when the honeybee received a stimulus signal on the right optic lobe, the flapping area of the right wings was closer to the abdomen than that of the wings on the left. The combination of differential flapping behavior and abdominal deflection leaded honeybee to possess an obvious tendency to fly to the stimulation side (the intact video is shown in Supplementary Movie 1). The behavioral responses were analogous to the attitude when honeybee perform yaw rotation during free flight [50]. Consequently, we considered the behaviors of abdominal deflection and differential flapping as the effective steering response, whose occurrence probabilities were calculated to judge the successful rates of honeybee steering control with different stimulation parameters.

Based on the above analysis of the response behaviors of tethered honeybees under steering flight initiation, we defined $n_{1}$ as the number of times the single honeybee produced only abdominal deflection to a specific stimulus parameter. In addition, we defined $n_{2}$ as the number of times the honeybee performed both abdominal deflection and differential flapping under a specific electrical stimulation. On this basis, proportion η was designated as $n_{1}/n$ (n=20, 10 on the left optic lobe, 10 on the right optic lobe), and proportion φ was referred to $n_{2}/n$.

The effect of the duty cycle on the steering initiation was firstly discussed. Five active honeybees participating in the experiments all received pulse electrical stimulation with a duty cycle of 20%-60%. In addition, the other parameters of the signals were identical with those of benchmark signal. However, the sequence of five parameters executing on various honeybees was different (Figure 2(c)), so as to rule out the effect of activity decline on steering feedback. According to the experimental results, the physiological activity of individuals did not wane significantly during the continuous steering stimulation, reflecting in the fact that the successful rate obtained by the later stimulation parameters did not decrease prominently (Figures 2(a) and 2(c)). More specifically, the successful rate of left steering initiation in individual 1 and individual 3 instantaneously drops to 0 in the second and fourth groups, respectively, which may be due to the destruction of the connection between electrode and left optic lobe. The stability of honeybee activity confirms that the locomotion control strategy of applying electrical signals to the brain optic lobes does not rapidly damage the athletic ability in a short period. Furthermore, the stability of honeybee vitality allows us to ignore the interference of vitality decline on the initiation successful rate of electrical stimulation parameters with different duty cycles.

In order to evaluate the overall success rate of each duty cycle, we averaged the proportion η and φ of the five honeybees under each stimulus parameter. The overall proportions are shown in Figure 2(d). According to the calculation results, the probability that honeybee produced abdominal deflection response under five stimulus duty cycles is higher than 70%. However, the probabilities of honeybee generating flapping response show a significant difference. Among the five tested stimulation parameters, the parameter with a duty cycle of 50% has the highest value in two proportions. Therefore, 50% is the optimal duty cycle for artificial steering control of honeybees and was determined as the benchmark duty cycle to participate in the following frequency optimization experiments.

**Figure 3:**
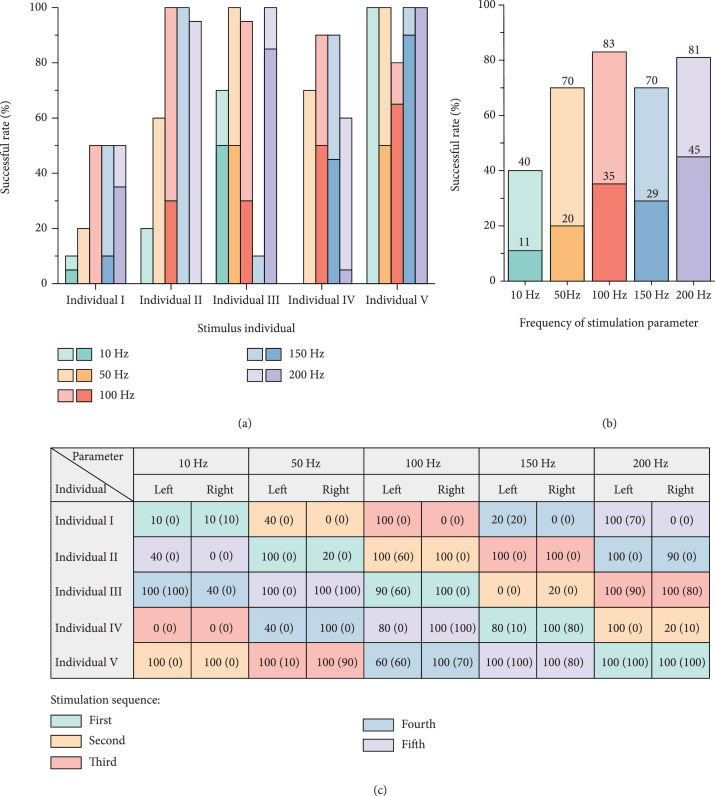
Successful rates of honeybee under electrical stimulations with different frequencies. (a) Successful rates of each honeybee to different electrical stimulus parameters. Among the two representative colors of each duty cycle, the lighter one represents η, the darker one represents φ. (b) The overall successful rates for each stimulus parameter in total five honeybees. In the bar chart of each duty cycle, the upper ratio is η, and the lower ratio is φ. (c) The sequence of electrical stimulation received by each individual and the respective successful rate of left and right initiation. The ratio outside the brackets is η, and the ratio inside the brackets is φ.

The impact of electrical signal frequency on stimulation successful rate was subsequently discussed. The frequencies of 10 Hz, 50 Hz, 100 Hz, 150 Hz, and 200 Hz were introduced to the participating in the steering flight initiation. Moreover, the optimal duty cycle of 50% as well as the voltage amplitude and duration of the benchmark parameters was appointed here for the electrical signals. The stimulation sequence for each frequency varied across the five individuals as well, as shown in Figure 3(c). The proportions of η and φ of each frequency achieved by five honeybees are shown in Figures 3(a) and 3(c), which did not appear a sudden decrease as the stimulation went on, so all the experimental results were introduced to acquire the overall response successful rate. According to calculation results shown in Figure 3(b), the response of abdominal deflection reached an extreme successful rate of 83% at the frequency of 100 Hz. However, the probability of obtaining a differentiated flapping response under the frequency of 200 Hz was significantly higher than that under 100 Hz. According to the existing research, abdominal flexion is indispensable for the insects to perform body rotation during free flying [51]. Consequently, the frequency of 100 Hz was identified as the optimal frequency for steering regulation of honeybees.

Noteworthily, the probability of honeybees performing flapping response under the stimulation with 100 Hz frequency and 50% duty cycle in the stimulation frequency optimization experiments (35%, shown in Figure 3(b)) was prominently lower than that of the same stimulation signal in the duty cycle optimization experiments. We speculate that this disparity might be due to the physiological activity difference of honeybees used in the two groups of optimization experiments.

### 3.2. The Effective Steering Torque under Unilateral Stimulation

The abdominal steering reflexes also occur when insect responses to visual or mechanical sensory stimuli and is acknowledged as an effective turning action of the insects [52–55]. In addition to the abdominal deflection, a distinct amplitude difference in the flapping appears when beetles make turning during muscular stimulation [42]. Zanker took Drosophila melanogaster as an example to analyze the influence of wing posture change and abdominal deflection on insect steering torque [49]. The abdominal deflection leads to yaw locomotion by displacing the line of action of aerodynamic drag force and increasing the friction difference between the two sides of the body [56, 57], while the variation of the wing beating amplitude directly generates yaw torque.

Since the honeybees tested in the optimization experiments were in an immobilized state, it is still uncertain whether the honeybee generates a swerving torque indispensable for the steering flight under the current locomotion control strategy. To further investigate the effectiveness of steering control strategy, we transformed the swerving torque of the honeybee into a visible suspension rotation through a magnetic levitation experimental system. The arrangement of the experimental system is shown in Figure 1(c). After immobilizing the honeybee, the system formed with suspension and honeybee achieved a force balance in the vertical direction under the magnetic field. If the honeybee generates a swerving torque capable of driving a body rotation, the suspension will rotate at the meantime. For the purpose of facilitating the comparison of suspension rotation before and after steering stimulation, we simultaneously photographed the suspension from the top and front view.

**Figure 4:**
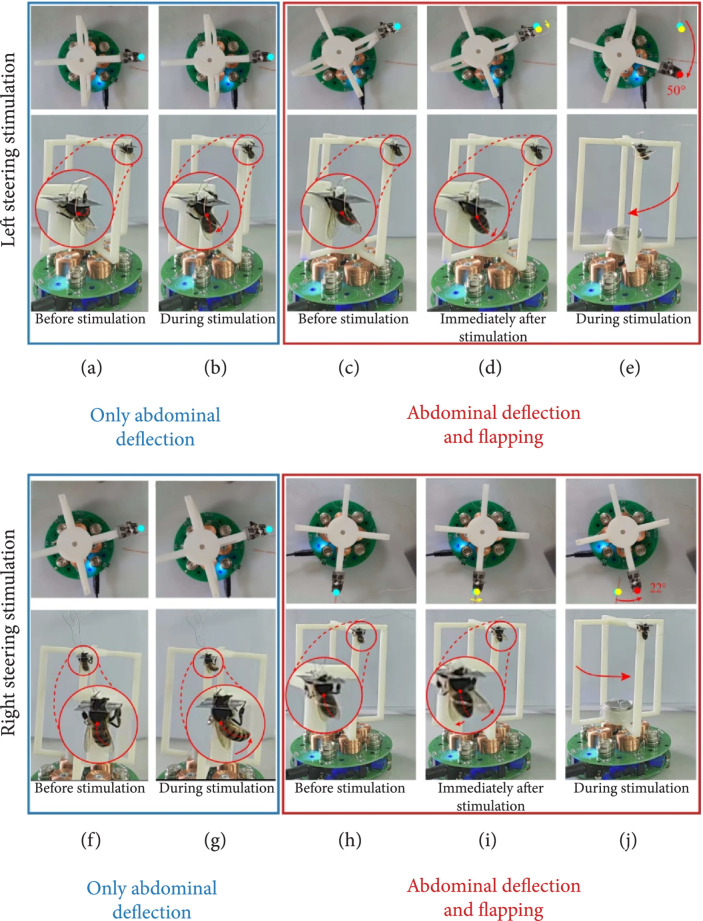
Steering torque verification through a magnetic levitation experimental system. The rotation of the suspension when the honeybee (a, b) (f, g) had only abdominal deflection response; (c–e) (h–j) had both abdominal deflection and flapping response under (a–e) left and (f–j) right steering stimulation. The blue, yellow, and red points represent the position of the honeybee before, immediately after, and during stimulation, respectively.

According to the photography results (the intact video is shown in Supplementary Movie 2), the position of suspension did not change evidently when the left steering stimulation only triggered an abdominal deflection (Figures 4(a) and 4(b)). Likewise, the steering stimulation on the right optic lobe that merely received a right-side abdominal deflection did not lead to a suspension rotation as well (Figures 4(f) and 4(g)). On the contrary, when the honeybee performed a flapping response to the steering electrical signal, the suspension acquired an obvious rotation at the meantime. Among them, the suspension rotation induced by the honeybee under a left electrical signal was in a clockwise direction (Figures 4(c)–4(e)), while the suspension rotated counterclockwise when the honeybee performed both abdominal deflection and flapping posture under the right steering stimulation (Figures 4(h)–4(j))). The rotation direction of the suspension was in line with the direction of the torque required in the left and right steering locomotion. The steering torques when the honeybee performed both abdominal deflection and differentiate flapping were obtained by calculating the moment of inertia of the system formed with suspension and honeybee, along with the rotational angular acceleration of the suspension. The left and right swerving torques generated in the steering process of honeybee shown in Figures 4(c)–4(e) and 4(h)–4(j) were 55.69 *μ*N·m and 69.91 *μ*N·m (the detailed calculation is shown in the Supplementary Material).

In the stimulation parameter optimization experiments, the honeybees flapped their left wings in the region close to the abdomen when received a left steering signal, while the right wings flapped closer to the head (Figure 2(b)). This asymmetrical flapping mode was not obviously reflected in Figure 4(d), because the honeybee lifted the body spontaneously to the right side before stimulation, making the flapping behavior constrained for the right wings. Actually, the body of the honeybee still had some movement space after the capsule was immobilized by the blades. The rotation of suspension caused by steering response clearly indicated that the honeybee generated a steering torque, thus confirming the effectiveness of the unilateral optic lobe electrical stimulation for honeybee steering control. It is notable that the steering response including only abdominal deflection induced indistinctive rotation of the suspension (Figures 4(a), 4(b), 4(f), and 4(g))). However, it does not imply that the abdominal deflection was not associated with steering torque generation, because the torque generated by abdominal flexion requires the combination with the airflow [49].

### 3.3. The Steering Control Effects of Unilateral Optic Lobe Stimulation on Crawling Honeybees

**Figure 5:**
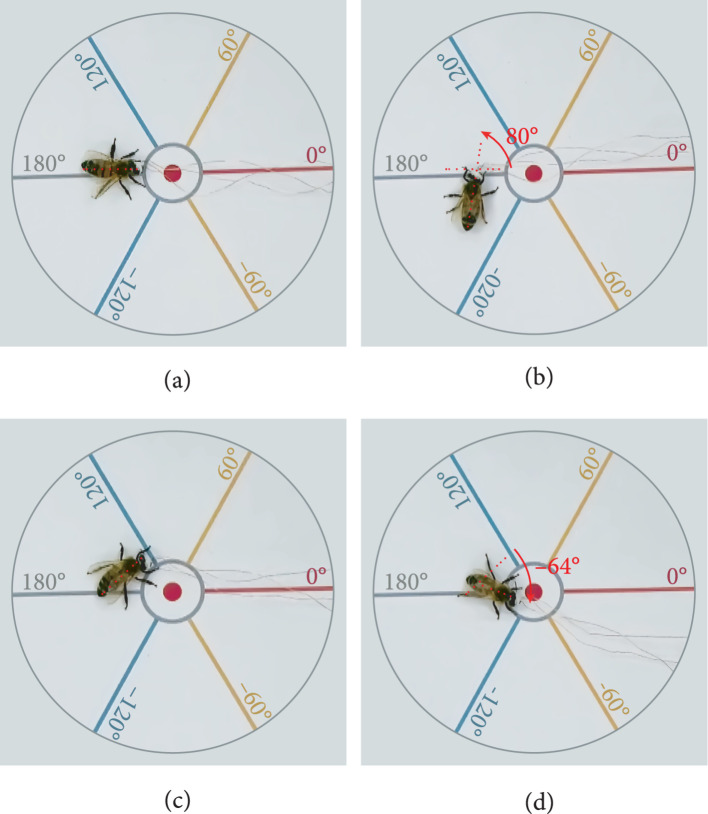
Steering control on the crawling honeybees. The body orientation of honeybee before (a) and after (b) left steering stimulation. The body orientation honeybee before (c) and after (d) right steering stimulation.

Since the steering behavior of honeybee under crawling status is absolutely different from that of honeybee under flying status, we applied electrical stimulation to the unilateral optic lobe of honeybees in crawling status to further verify the steering control effect. The parameters used here were consistent with the optimal steering control parameters determined in 3.1, i.e., 3 V, 100 Hz, 50%, and 1 s. As shown in Figure 5, we take the attitude that the significant body rotation (greater than the angular deviation threshold of 20 degree) to the side of electrical signal was an effective behavior response after receiving stimulation (the turning behavior is shown in Supplementary Movie 3). Unilateral optic lobe stimulation initiated a left turn crawling and a right turn crawling 27 times and 23 times, respectively (N=5, n=100, 50 for left and 50 for right). In addition to the successful steering stimulus, the behavioral response of honeybees in the remaining 50 failed trails is shown in Table 1. In the total of 28 failed trials with trembling response, 15 failures were due to the detachment of the foot sole from the circular disk, thus affecting the crawling movement. This phenomenon was caused by the contact between the front paw of honeybee and the electrodes immobilized on the head. The retreat response may due to the honeybee mistaking the stimulating signals for a mechanical contact, thus resulting in an escape reaction. Partial crawling individuals also performed flapping response after receiving stimulation, subsequently producing a low-altitude flight and a rapid departure from the initial position (Supplementary Movie 4). In the 9 failed trials with forward flight response, there was no obvious angular deviation between the honeybee body orientation before and after stimulus. Therefore, the response in these 9 trials was considered forward flight instead of steering. Overall, the electrical stimulation on the unilateral optic lobe can trigger the locomotion response of the crawling honeybees with a high probability, and its average successful rate in initiating a left rotation and right rotation is 54% and 46%, respectively.

**Table 1:** The behavioral response of honeybees in the failed trials.

Behavioral response	The number of occurrences
Trembling	28
Retreat	9
Forward flight	8
No response	5

## 4. Discussion

We employed the binomial test to determine the sample size significance of the electrical stimulation parameters of 3 V, 100 Hz, 50%, and 1 s on the steering control of tethered and crawling honeybees. The p value of 0.05 and the excepted successful rates of 75% (left steering control for tethered honeybee), 78% (right steering control for tethered honeybee), 32% (left rotating control of crawling honeybee), and 25% (right rotating control of crawling honeybee) were used to test the significance of successful rates. The average successful rates were 83%, 90%, 54%, and 46% for these four conditions, which were higher than the expected successful rates (p=0.01, for tethered honeybee, N=10, n=200, 100 for left and 100 for right; for crawling honeybee, N=5, n=100, 50 for left and 50 for right). The test results indicated that the steering stimulation strategy was significantly successful under the above criteria.

Honeybees in tethered and crawling states perform a distinct behavioral response to the electrical stimulation on the unilateral optic lobe. The effective responses of tethered honeybee include abdominal deflection and differential flapping, which are comparable to the turning locomotion of beetles elicited by the same steering control strategy [44]. Based on the optimization of stimulation parameters, the successful rate of the steering control for tethered honeybees has been improved compared with Zhao et al. [47].

The statistical results demonstrated that the successful rate of steering stimulation for honeybee in suspended state is significantly higher than that in crawling states. According to the analysis of failures in steering control on crawling honeybees, it is obvious that the desorption of the footpad caused by the contact between the paws and electrodes is one of the main reasons that affect the steering crawling. Therefore, we reasonably speculate that the appropriate arrangement of the electrodes connecting the head capsule and artificial stimulation module can reduce the possibility of contact between honeybee claws and electrodes, thereby improving the successful rate of steering control for crawling honeybees.

In the steering control of crawling honeybees, although the electrodes connected to the head capsule caused the rejecting behavior, manifesting as the contact between honeybee claws and electrodes, the honeybee still maintained the voluntary locomotion ability under the existing electrode arrangement. Combined with the steering torque verified by the magnetic levitation experimental system, we conclude that the existing locomotion control strategy can achieve a steering regulation effect on free-flying honeybees. To date, researchers have not realized the wireless flight control of small insects represented by honeybees. One of the main obstacles is the confined load capacity, limiting the design and manufacture of wireless locomotion control devices. In the future, we will make efforts to develop the wireless locomotion regulation module that is more in line with the honeybee load characteristics.

## 5. Conclusion

A highly efficient and reliable locomotion control strategy is well accepted as the premise to ensure the accomplishment of preset motion trajectories and assignments for cyborg insects. In this study, we validate the effectiveness of steering flight control based on unilateral optic lobe electrical stimulation for both tethered honeybees and crawling honeybees. To further improve the successful rate of steering control, we optimized the duty cycle and frequency of the stimulation parameters.

The experimental results of stimulation parameter optimization indicate that the electrical signal with 3 V in amplitude, 100 Hz in frequency, 50% in duty cycle, and 1 s in duration was competent to induce the steering action for tethered honeybee with the highest successful rate, which was suitable for the steering control of crawling honeybees as well. Moreover, the successful rate of steering initiation of crawling honeybees will achieve a further improvement by optimizing the electrode arrangement between the stimulation module and the head capsule.

The locomotion control technique of optic lobe electrical stimulation was generally applied as a supplementary strategy other than motor muscle electrical stimulation to enhance the successful rate of flight initiation of cyborg beetles [45, 58]. However, the steering initiation was still accomplished through steering muscle stimulation. Our study has fully confirmed the promising applicability of electrical stimulation applied to the unilateral optic lobe for steering control, which is beneficial to reduce the electrode implantation on cyborg insects, ensure the successful rate, and maintain the functional integrity of cyborg insect biological tissues.

## Data Availability

Data supporting the findings of this study are available in the main text or the supplementary information.

## Supplementary Materials

Supplementary Materials Supplementary 1. Movie S1: left and right steering control to the tethered honeybee. Supplementary 2. Movie S2: the rotation of the suspension when honeybee performs different steering response. Supplementary 3. Movie S3: left and right steering control of the crawling honeybee. Supplementary 4. Movie S4: crawling honeybee performs flapping response under steering control. Supplementary 5: the design of the magnetic levitation experimental system and the calculation of the steering torque.
