# Supplementary material for: Experimental Verification on Steering Flight of Honeybee by Electrical Stimulation
Source: Cyborg Bionic Syst. 2022 Jul 21;2022:9895837. doi: 10.34133/2022/9895837 (PMC11780726; doi:10.34133/2022/9895837)
Supplement: Supplementary Materials — Supplementary 1. Movie S1: left and right steering control to the tethered honeybee. Supplementary 2. Movie S2: the rotation of the suspension when honeybee performs different steering response. Supplementary 3. Movie S3: left and right steering control of the crawling honeybee. Supplementary 4. Movie S4: crawling honeybee performs flapping response under steering control. Supplementary 5: the design of the magnetic levitation experimental system and the calculation of the steering torque. [file 9895837.f1.zip › 9895837.f1/Supplementary Material.docx]

Supplementary Materials

The design of the magnetic levitation experimental system

The experimental system was designed based on the push-down magnetic levitation system, which mainly included suspension magnets, electromagnetic poles, electronic coils, stator magnets, and circuits (Figure S2). The three suspension magnets were made of ferrite permanent magnet material, and the electromagnetic poles inside the coils were made of electrical pure iron (24 mm diameter; 3 mm thickness. One of the three magnets can be replaced by a ring magnet with an inner diameter of 10 mm, an outer diameter of 18 mm and a thickness of 3 mm). The four coils were divided into two groups, of which the first and third coil were in one group, and the second and fourth coil were in another group. The coils in one group were connected in reserve series. Therefore, when the coils were energized, the coils in one group generated opposite magnetic fields and created suction and repulsion on the suspension, respectively. For example, when the suspension got close to the coil 1, the coil 1 produced a repulsive force to the suspension, and the coil 3 attracted the suspension. Similarly, when the suspension approached coil 3, coil 3 repelled the suspension, and coil 1 drew the suspension. The force directions of the two sets of coils on the suspension were perpendicular, thereby realizing the position locking of the suspension. The suspension magnets were fixed in the bottom of the suspension (Figure S1).


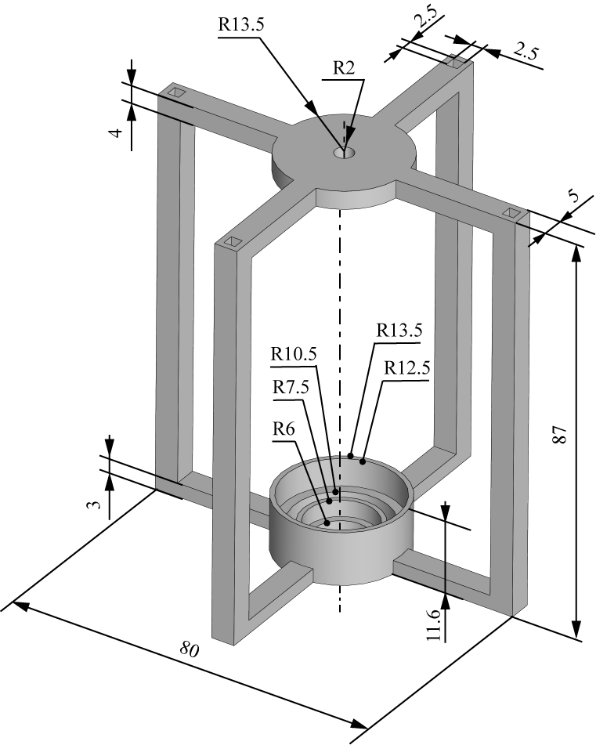


**Figure S1. The shape and size of the suspension.** The dimensions are in mm.


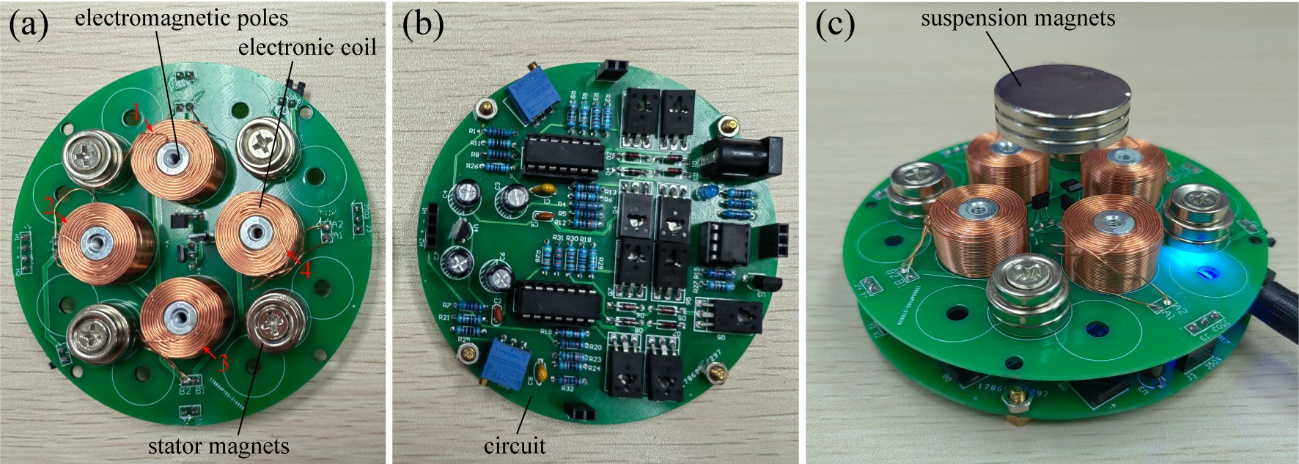


**Figure S2. The composition of the magnetic levitation system.** (a) The upper layer of the system. (c) The lower layer of the system. (c) The overall structure of the system.

In order to change the force direction of the coil on the suspension according to the suspension position, the circuit controlling the same group of coils should equip the function of switching the current direction. This function was realized by an H-bridge circuit composed of 8 triodes, as shown in Figure S3.


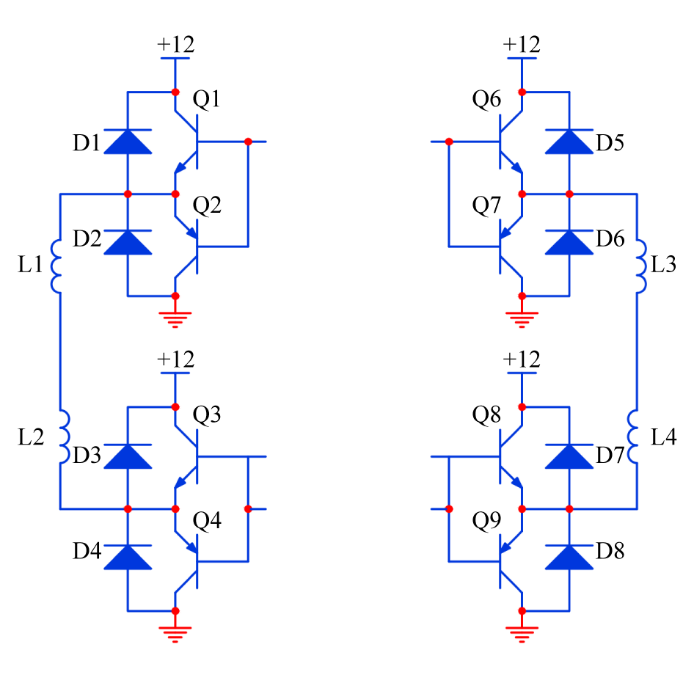


**Figure S3. The circuit for coil current direction switching.**

The position deviation of the suspension was judged by the analog voltage output of a Hall sensor (AH49E), whose power supply voltage was set to 5 V. To meet the power supply of the Hall sensor, we used the voltage regulator (TL431) to reduce the laboratory power supply voltage. The step-down circuit is shown in Figure S4.


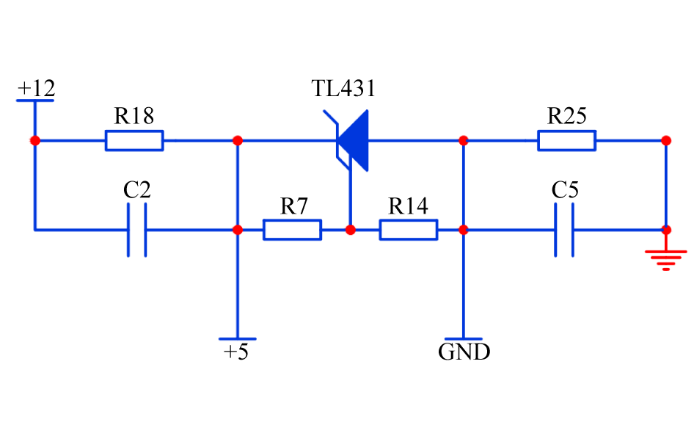


**Figure S4. The step-down circuit is shown in Figure S3.**

The analog voltage range of the Hall sensor output was 0-5 V. We use the potentiometer to adjust the reference voltage of the Hall sensor to 2.5 V. When the sensor output was lower than the reference voltage, it means that the suspension magnets were biased to the left, then the H-bridge transistor was turned to energize the coil; when the sensor output was higher than the reference voltage, it means that the suspension was biased to the right, and the H-bridge transistor was turned on to energize the coil in reverse. The sensor output was amplified by the operational amplifier (LM324) in two ways to realize the forward and reverse conduction of the H-bridge transistor.

For the purpose of reducing the power consumption of the system, a circuit was supplemented to ensure that the circuit did not work when there was no suspension (Figure S5). Hall sensor (J3) was used to judge the conduction of the triode. When the sensor output to the positive end of the voltage comparator (LM393) was lower than the negative end, the triode was turned on, and vice versa.


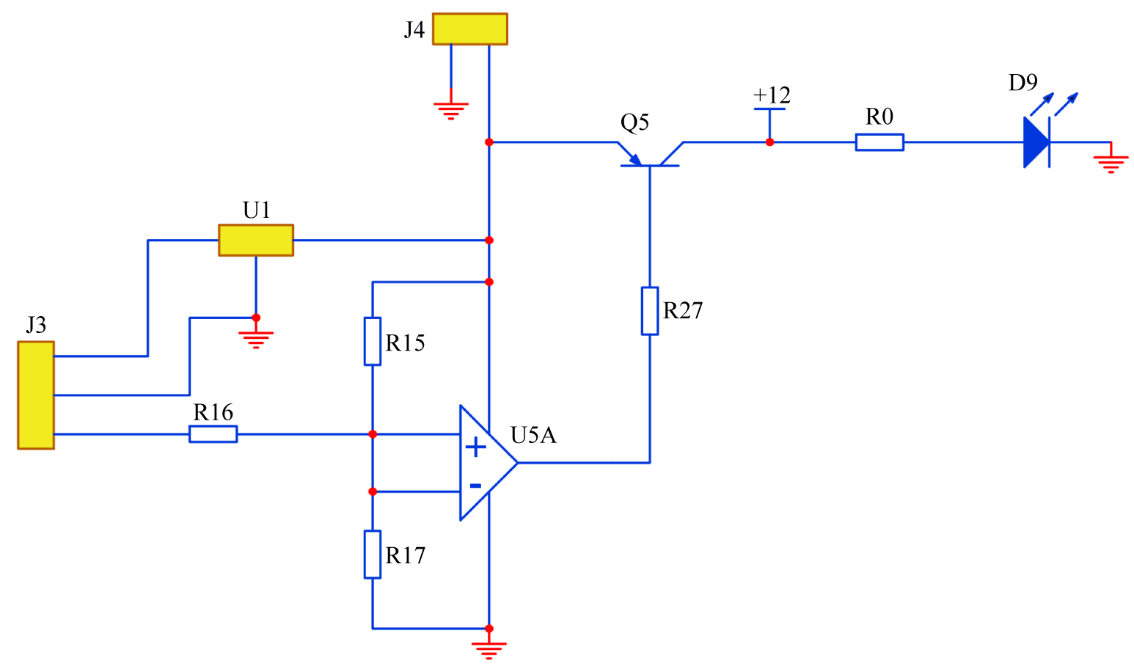


**Figure S5. The circuit for reducing the power consumption.**

Combining the above circuit modules, the final circuit diagram is shown in Figure S6, and the model parameters of the circuit components are shown in Table S1.


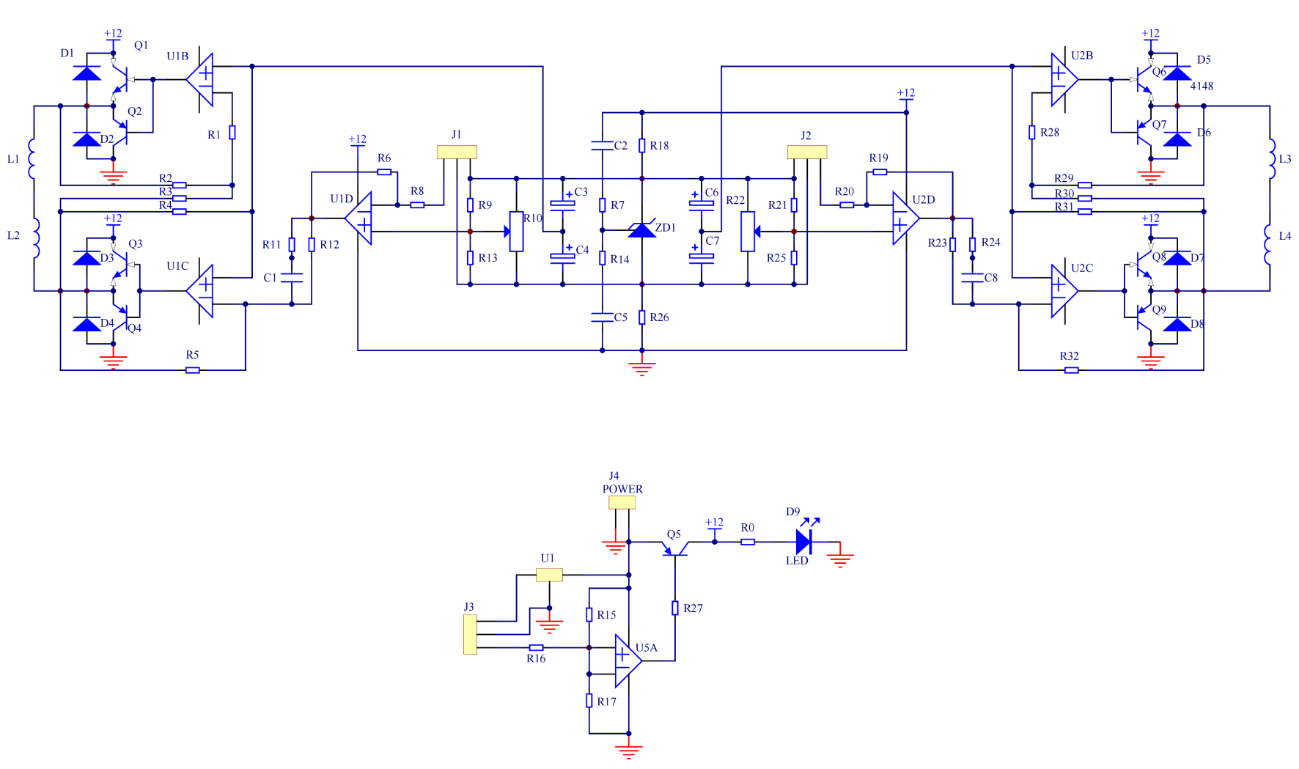


**Figure S6. The overall control circuit of the magnetic levitation system.**

**Table S1. The model parameters of the circuit components**

| Electronic component type | Component code | Model and parameter |
| --- | --- | --- |
| resistor | R1, R28 | 430 K |
|  | R2, R29 | 1.5 K |
|  | R3, R30 | 1.2 K |
|  | R4, R31 | 330 K |
|  | R5, R8, R10, R20, R22, R32 | 10 K |
|  | R6, R19 | 130 K |
|  | R7, R14, R16, R17 | 5.1 K |
|  | R0, R9, R11, R13, R21, R24, R25, R27 | 1 K |
|  | R12, R23 | 3.3 K |
|  | R15 | 20 K |
|  | R18, R26 | 180 |
| capacitor (non-polar) | C1, C8 | 105 |
|  | C2, C5 | 104 |
| capacitor (polar) | C3, C4, C6, C7 | 220 uF |
| potentiometer | D1 | TL431 |
| switching diode | D1-D8 | 4148 |
| voltage regulator | ZD1 | 78L05 |
| operational amplifier | U1B, U1C, U1D, U2B, U2C, U2D | LM423 |
| voltage regulator | U5A | LM393 |
| PNP transistor | Q2, Q4, Q5, Q7, Q9 | 772 |
| NPN transistor | Q1, Q3, Q6, Q8 | 882 |
| light-emitting diode | D9 |  |
| Hall sensor | J1-J3 | AH49E |

The calculation of the steering torque

Before steering initiation, the suspension was manually stabilized to avoid the rotation and keep stationary. It was confirmed that, the swerving torque existed if the suspension rotated around the central axis while the honeybee performed a steering posture under stimulation. Here we mainly calculated the steering torque when honeybee produced both abdominal deflection and differential flapping under stimulation.

The steering torque T was calculated by Eq. 1,

|  | $T=J\cdot a$ | (1) |
| --- | --- | --- |

where *J* was the total moment of inertia of the suspension, permanent magnets, blades, and honeybee, *a* was the rotational acceleration of the suspension.

Since the weights of the blades (400 mg) and honeybee (100 mg) were much lower than those of suspension (16.20 g) and magnets (23.09 g), we ignored the effect of blades and honeybee on the total moment of inertia *J*. The dimension of the suspension is shown in Figure.S1. The suspension was decomposed into several slender rods, discs, and thin-walled cylinders to obtain its overall moment of inertia *J_s_*. After the moment of inertia of the suspension and the magnet were obtained, the total moment of inertia J was calculated by Eq. 2,

|  | $J=J_{s}+J_{m}$ | (2) |
| --- | --- | --- |

where the moment of inertia of suspension *J_s_* was 1.26865×10^-4^ Kg·m^2^, and the moment of inertia of magnet *J_m_* was 1.597×10^-6^ Kg·m^2^.

The suspension rotated in a clockwise direction when the honeybee produced both abdominal deflection and flapping behavior under left steering initiation. The rotation angle is shown in Figure 4e. Likewise, the suspension rotated counterclockwise when honeybee produced both abdominal deflection and flapping behavior under right steering initiation (Figure 4j). The durations of the suspension rotating in two directions recorded by the DSLR camera were 2.013 s and 0.7056 s. We assumed that the angular acceleration was uniform throughout the entire process of suspension rotation elicited by the honeybee steering response. Based on the assumption, the clockwise steering acceleration *a_L_* and the counterclockwise steering acceleration *a_R_* of the suspension in the rotating period shown in Figure 4c-e and Figure 4h-j were 0.4335 rad/s and 0.5442 rad/s. Therefore, the left and right steering torque generated by the honeybee were 55.69 μN·m and 69.91 μN·m, respectively.

Figures S1 to S6#

Tables S1 #

Movies S1 to S4#
